# Model of SNARE-Mediated Membrane Adhesion Kinetics

**DOI:** 10.1371/journal.pone.0006375

**Published:** 2009-08-03

**Authors:** Jason M. Warner, Erdem Karatekin, Ben O'Shaughnessy

**Affiliations:** 1 Jason M. Warner, Department of Chemical Engineering, Columbia University, New York, New York, United States of America; 2 Erdem Karatekin, Institut de Biologie Physico-Chimique, Centre National de la Recherche Scientifique UPR 1929, Paris, France; 3 Ben O'Shaughnessy, Department of Chemical Engineering, Columbia University, New York, New York, United States of America; German Cancer Research Center, Germany

## Abstract

SNARE proteins are conserved components of the core fusion machinery driving diverse membrane adhesion and fusion processes in the cell. In many cases micron-sized membranes adhere over large areas before fusion. Reconstituted *in vitro* assays have helped isolate SNARE mechanisms in small membrane adhesion-fusion and are emerging as powerful tools to study large membrane systems by use of giant unilamellar vesicles (GUVs). Here we model SNARE-mediated adhesion kinetics in SNARE-reconstituted GUV-GUV or GUV-supported bilayer experiments. Adhesion involves many SNAREs whose complexation pulls apposing membranes into contact. The contact region is a tightly bound rapidly expanding patch whose growth velocity 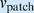 increases with SNARE density 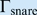. We find three patch expansion regimes: slow, intermediate, fast. Typical experiments belong to the fast regime where 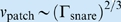 depends on SNARE diffusivities and complexation binding constant. The model predicts growth velocities 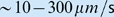
s. The patch may provide a close contact region where SNAREs can trigger fusion. Extending the model to a simple description of fusion, a broad distribution of fusion times is predicted. Increasing SNARE density accelerates fusion by boosting the patch growth velocity, thereby providing more complexes to participate in fusion. This quantifies the notion of SNAREs as dual adhesion-fusion agents.

## Introduction

In cells the controlled delivery of materials packaged by membrane-bound organelles and vesicles is achieved by membrane fusion. SNARE proteins are involved in most intracellular eukaryotic fusion processes [1] and have been termed the fusion “workhorses” [2] and the “minimal fusion machinery” [3]. SNAREs dock membranes in preparation for fusion: a t-SNARE in one membrane binds its cognate v-SNARE partner in the apposing membrane, forming a SNARE complex as their cytoplasmic domains combine into a four-helix bundle [4]. For example, in the presynaptic membrane syntaxin and SNAP25 form a t-SNARE acceptor complex that binds the v-SNARE synaptobrevin provided by the synaptic vesicle [1]. The resulting helical bundle contains one helix from syntaxin, one from synaptobrevin and two from a single SNAP25 molecule [4]. The crystal structure of the SNARE complex suggests that its complete assembly pulls membranes into close contact [4].

It has been postulated that SNAREs are dual adhesion-fusion agents. Subsequent to bringing membranes into intimate contact, it has been proposed that SNAREs trigger fusion [3] though additional proteins are thought to be involved [2]. The SNARE complex is highly stable, suggesting assembly may release work to drive fusion [2]. However, the role of SNAREs in the fusion step remains unsettled. Fusion was prevented or reduced when *trans* complex assembly was blocked in PC12 [5] and chromaffin cells [6], but not for yeast vacuoles [7] or sea urchin egg vesicles [8].

Identifying and quantifying the role played by SNAREs is challenging because the complex cellular fusion machinery involves many components. A substantial body of *in vitro* studies [3], [9]–[14] has sought to isolate their contribution by reconstituting SNAREs into synthetic small unilamellar vesicles (SUVs) and supported bilayers (SBLs). These studies illuminated both SNARE-mediated adhesion and fusion mechanisms. One study concluded that only one SNARE complex is required for SUV-SBL docking [12]. Liu et al [10] found diffusion-limited docking rates, i. e. a SUV is almost instantly captured by nearby SBL t-SNAREs. In reconstituted synaptic SNARE systems typical measured fusion times are ∼10 min and ∼10 s in, respectively, SUV-SUV [3], [9] and SUV-SBL [11], [12] systems.

SUV studies have contributed significantly to current understanding of SNARE function. Nonetheless questions remain as to the cellular relevance of *in vitro* mechanisms. Typical measured fusion times greatly exceed the ∼1 ms required for synaptic vesicle fusion [15]. Though ∼25 ms has been achieved *in vitro*, SNAP25 was not required [10] suggesting the fusion may have been non-specific, mediated by weak syntaxin-synaptobrevin binding [1]. One study reported SNAREs did not trigger SUV fusion alone but could promote fusion of PEG-aggregated SUVs [13]. A possible complication is that *in vitro* fusion events may result from the small sub-population of vesicles rendered inherently unstable by particularly high curvature and SNARE∶lipid ratios [14].

Most *in vitro* studies have used ∼50-nm SUVs, appropriate to processes such as synaptic transmission where 50-nm vesicles fuse with the presynaptic plasma membrane ∼1 ms after Ca^2+^ stimulation [2], [16]. However micron-sized membranes are involved in many processes such as adhesion and fusion of yeast vacuoles lasting tens of seconds [2], [17]. These membranes contain many SNAREs and adhere over areas 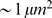 before fusion [17]. Other examples include large vesicle (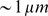 diameter) trafficking [18], lysosome (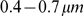) fusion [19] and exocytosis of acrosomal vesicles (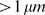) [20] and cortical granules (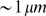) [21]. Flipped SNAREs mediate cell-cell adhesion over areas 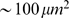 [22].

To mimic large membrane cellular fusion systems it is natural to turn to *giant* unilamellar vesicles (GUVs). Studies have begun to realize the potential of SNARE-reconstituted GUVs as model *in vitro* systems which may reveal mechanisms of adhesion and fusion of micron-scale membrane compartments. Bacia et al [23] reconstituted labeled SNAREs into 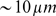 GUVs and showed the SNAREs bound with their solubilized cognates. SNARE spatial distributions were visualized and their in-membrane diffusivities measured. Another recent study showed that t-SNARE-reconstituted large unilamellar vesicles (LUVs) adhered and fused with v-SNARE GUVs [24]. On average at least 2 LUVs were bound to each GUV and were mobile on the GUV surface. Lipid mixing kinetic data indicated a fusion rate 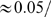
min per 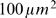 of GUV membrane [24]. Assuming irreversible docking and taking GUV diameter 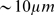 this suggests LUVs remained adhered for at least 14 min on average before fusion.

**Figure 1:**
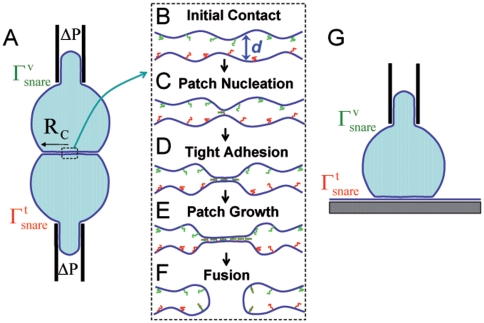
Schematics of proposed *in vitro* experiments following SNARE-mediated adhesion and fusion kinetics. (A) GUVs reconstituted with t-SNAREs ( *red*, surface density 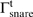) and v-SNAREs ( *green*, surface density 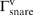) are aspirated into micropipettes and pushed together generating a contact zone of radius 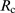. Aspiration pressure 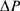 controls membrane tension 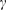. Due to GUV size, patch evolution and fusion may be followed by various optical microscopy techniques in real time. ( *b–f*) Blow-up of box in (A). (B) Tension determines the initial membrane separation in the contact zone, 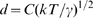; below this separation, membranes are strongly repulsive due to entropic membrane fluctuations (omitted in (A) for clarity). Complexation is hindered because 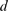 is larger than SNARE reach. (C) A membrane fluctuation brings SNAREs together, nucleating tight adhesion at time 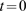. (D,E) Growth of the adhesion patch at velocity 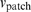. Complex assembly is facilitated by closeness of membranes in the patch. (F) SNARE complexes trigger fusion at time 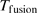. (G) Similar to (A) but t-SNARE membrane is now a SBL. Reflection interference contrast microscopy (RICM) is an ideal technique to follow patch areal growth.

In this paper we develop a model of SNARE-mediated adhesion kinetics in controlled SNARE-reconstituted GUV-GUV or GUV-SBL systems. We discuss experiments where such adhesion kinetics could be followed and GUV membrane tension and SNARE surface densities precisely controlled (see Proposed Experiments and Fig. 1). Since contact areas are large, many SNAREs may be involved and the ensuing adhesion and fusion kinetics may reflect collective behavior qualitatively distinct from that in SUV systems. Our model predicts that after first membrane contact a growing adhesion patch develops as increasing numbers of SNARE complexes bridge the membranes (see Fig. 1). The adhesion kinetics and SNARE density profiles depend on membrane tension and initial SNARE densities in the membranes and quantitatively reflect basic SNARE properties such as in-membrane diffusivities and the SNARE complexation rate constant 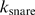.

Though adhesion is our focus, we briefly consider fusion kinetics. The formation of large many-SNARE adhesion domains may lie on the pathway to large membrane fusion (Fig. 1F). There is evidence for this sequence in LUV-GUV systems [24], yeast vacuole fusion [17] and intercellular fusion mediated by flipped SNAREs [22]. Since it is unknown if SNAREs work collectively, to model fusion kinetics we invoke the simplest assumption that each SNARE complex *independently* triggers fusion with a certain mean waiting time. We will show this leads to an effective coupling between adhesion and fusion: the larger the adhesion patch the more assembled SNARE complexes and so the greater the net fusion probability per unit time and the smaller the overall mean fusion waiting time.

A SNARE and its cognate partner is an example of a biosticker-ligand pair (albeit one which may additionally catalyze fusion). Surface adhesion by other biosticker systems was observed to progress by growth of tightly bound patches, including GUV-substrate adhesion [25]–[28] and cell spreading [29]–[31]. Boulbitch et al [25] found two regimes of adhesion patch growth between ligand-bearing GUVs and integrin-covered substrates: at low ligand densities patch radius 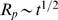 after time *t* in accord with a predicted ligand-diffusion-limited regime while at high densities 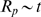 consistent with a predicted binding-kinetics-limited regime. Cuvelier and Nassoy [26] found 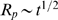 in adhesion of streptavidin-coated GUVs to biotin substrates at low streptavidin densities while at saturating densities patch velocity decreased exponentially in time. They modeled the two regimes as, respectively, diffusion-controlled and viscous dissipation-limited.

Since SNAREs and their complexes are apparently *mobile* [24] SNARE-mediated adhesion kinetics presumably differ fundamentally from those discussed above where one of each sticker-ligand pair was *immobilized* on a substrate. Thus different mathematical models are necessary to describe SNARE adhesion. Mobile complexes may exert 2D osmotic pressure tending to enlarge a patch. De Gennes, Puech, and Brochard-Wyart [32] modeled this class of situations and found patch growth is initially binding-kinetics-limited with 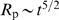 and then attains constant speed in steady state, 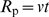. Assuming *uniform* complex density in the patch they predicted growth velocity *v* increases as the 3/2 power of receptor and sticker density. In this paper we explicitly calculate SNARE density profiles and show that in fast growing patches the complex density is in fact severely depleted at the boundary. Osmotic pressure and growth rate are thus diminished and a different power law results. Using properties taken from the literature we find typical SNARE systems belong to this fast growth regime.

In our model the origin of adhesion patch growth is that the initial tension-dependent mean membrane separation in the GUV-GUV or GUV-SBL contact zone (Fig. 1B) normally exceeds the reach of cognate SNAREs (∼8 nm [33]). Hence the first complexation event is a slow process, requiring SNAREs to connect across this large gap (Fig. 1C). Once achieved, however, the tight membrane contact in this location accelerates further SNARE binding (Fig. 1D,E). Thus a patch grows, driven by SNARE complex osmotic pressure and resisted by viscous drag. The force balance results in a growth speed 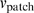.

In the Discussion the possible relevance of these results to cellular fusion pathways is addressed. Tight SNARE adhesion is preceded by loose binding by tethering factors. Given typical tether sizes (e.g. ∼30 nm for the exocyst [34]) the initial membrane separation may exceed SNARE reach which for large membranes may lead to self-promoting SNARE adhesion patches similar to those predicted here for *in vitro* systems.

### Proposed experiments

Before introducing the model we first describe proposed experiments yet to be performed which can test our predictions. The model directly describes *in vitro* experiments of the type shown in Fig. 1. One GUV is reconstituted with t-SNAREs (surface density 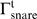) while the second GUV or the SBL is reconstituted with cognate v-SNAREs (density 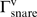). GUV membrane tensions *γ* would be controlled by micropipette suction pressure [35] or by using heavy GUVs in the GUV-SBL setup [36].

The total GUV-GUV or GUV-SBL contact area 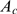 is controlled by pressing the surfaces into contact or by a balance of gravitational forces and membrane tension in the heavy GUV-SBL setup [36]. In this contact zone, the initial membrane separation *d* is controlled by the applied pressure and the surface tension *γ*, the latter set by micropipette suction. Repulsive electrostatic forces overcome non-specific van der Waals adhesion provided the fraction *Φ* of negatively charged lipids is sufficiently large (Evans found the requirement 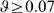 in physiological salt solutions, 0.1 M NaCl [37]). The mean separation is then governed by entropic membrane undulations. Theory predicts [38], [39]

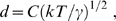
(1)where 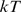 is the thermal energy at temperature *T* and *C* depends only logarithmically on tension and applied pressure. This result is valid for sufficiently low tensions where *d* exceeds the range of electrostatic, van der Waals, and hydration forces which decay rapidly with separation. The result follows, for example, if one sets van der Waals forces to zero in ref. [39].

An adhesion patch is expected to nucleate since undulations occasionally bring cognate SNAREs together. In the GUV-SBL arrangement the subsequent patch growth kinetics can be monitored using reflection interference contrast microscopy (RICM, see Fig. 1G). The large dimensions of GUVs enable other optical microscopic techniques [23], [35], [40].

## Methods

### Model

#### Initial conditions

We model GUV-GUV or GUV-SBL adhesion in experiments described in the previous section. (The “vesicle-vesicle” language will be used.) For simplicity the symmetric case is assumed: both vesicles have equal numbers of SNAREs per unit area in their respective membranes, 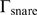. Before complexation the vesicles are separated by a distance *d* exceeding the SNARE complexation reach 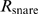 over a large contact area 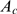 (Fig. 1).

#### Objectives of model

The first SNARE complex assembles at time 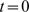, nucleating a tightly bound adhesion patch whose radius 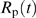 subsequently grows as more complexes form (Fig. 1C–E). The patch is self-promoting: once nucleated, it provides a reduced intermembrane separation zone where complexation is easier.

Our interest is steady state patch growth where the velocity 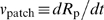 is constant. Our principal goal is to predict how the steady state 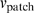 depends on vesicle SNARE densities 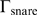, membrane tensions *γ*, SNARE diffusivities and the complexation rate constant 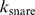. The rate constant is a fundamental SNARE property measuring kinetics of complexation “reactions” characterized by “capture radius” 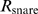.

To calculate 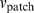 the steady state SNARE profile 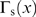 in each vesicle must be determined. Deep inside the patch this falls to zero due to complexation, while far from the patch this tends to the initial value 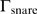. The SNARE complex profile, 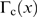, vanishes outside the patch by definition; once a complex forms it is trapped in the patch by the connection created.

#### Patch growth velocity results from balance of osmotic pressure and drag forces

Patch growth is driven by the 2D osmotic pressure 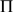 of SNARE complexes [32], [41]. Pressure is mediated by the membrane diffusivity 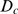 of complexes. Assuming ideal gas statistics, the osmotic pressure is
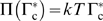
(2)where 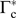 is the complex density at the patch boundary.

**Figure 2:**
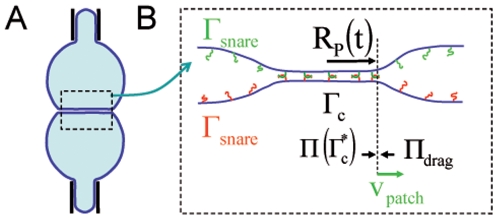
Model of SNARE-mediated adhesion: schematic of patch growth. Following nucleation SNARE complexes (density 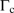) assemble inside a tightly bound adhesion patch (radius 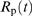). t-SNAREs ( *red*, surface density 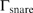) and v-SNAREs ( *green*, density 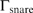) can bind only inside the patch where the membrane separation is sufficiently small. Complexes near the patch boundary at density 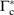 exert 2D osmotic pressure 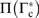 on the boundary. This drives patch growth at velocity 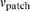 determined by a balance of 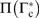 and the velocity-dependent resistive force per unit length 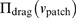.

In steady state the outward osmotic pressure is balanced by dissipative drag forces 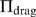 opposing growth (see Fig. 2). These dissipative forces are of complex origin and presumably include dissipation due to expulsion of intermembrane fluid accompanying patch growth. Thus we adopt a simple linear relation with drag coefficient 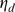 whose dimensions are viscosity and whose value is in principle available from experimental measurement of patch growth,
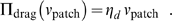
(3)


As fluid is not expected to significantly penetrate the patch, dissipation occurs primarily in a narrow band along its boundary. The coefficient 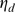 measures dissipation per unit length of patch boundary and is independent of patch size. More generally 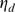 is the local slope of the drag-velocity relation. The steady state patch velocity satisfies the force balance 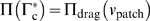 (see Fig. 2), yielding a linear dependence on the complex density at the patch boundary,
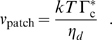
(4)


Since a patch grows within contact zone of area 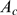 and patch area is much less than 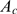 (Fig. 1) patch growth does not increase vesicle surface area and elevate surface tension which would resist growth.

#### Equations governing steady state density profiles

To obtain the patch velocity 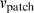 from eq. 4 we must determine the complex density at the boundary, 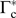. This can only be obtained by calculating the steady state complex density profile in space 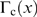, which in turn depends on 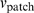. Thus the profile equations are solved simultaneously with eq. 4 as a dynamic boundary condition at the patch edge (see below).

For simplicity we assume the two SNARE types have equal diffusivities 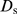. The diffusivity of complexes is expected to be smaller, 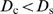. A cognate pair can complex only if both SNARES diffuse into the patch. Complexation then follows 2nd order “reaction” kinetics characterized by a 2D rate constant 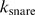. A 2D framework is valid provided the membranes are sufficiently closely adhered. This is satisfied for typical experimental SNARE densities (see Supplementary Material S1). Irreversibility is assumed since the SNARE complex is highly stable [42].

We seek equations governing the steady state densities. In the region close to the patch boundary densities will change substantially as a function of position. Provided the patch radius is much larger than the size of this region, the situation becomes approximately 1D in the direction orthogonal to the patch boundary, *x*, and the far field boundary conditions are in effect at 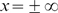. During steady state growth the density fields are unchanging in a frame of reference moving with the boundary. We name this density field for the SNAREs 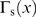 where *x* is distance from the boundary, and similarly 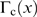 for the complexes. (Note the SNARE density profile 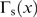 is the same in each vesicle by symmetry.) In Supplementary Material S1 it is shown these obey
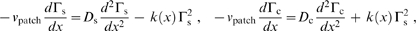
(5)where
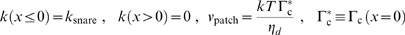
(6)and the boundary conditions are
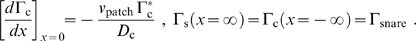
(7)


Each of eqs. 5 consists of a convective term proportional to 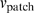, a diffusive term involving the relevant diffusivity, and a 2nd order complexation “reaction” term of magnitude 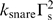 within the patch (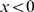). For a given SNARE density 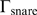, the task is to solve eqs. 5, 6, and 7. Choosing 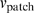 arbitrarily in eq. 5 would generate density profiles which would then define a velocity 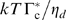; the correct patch velocity choice satisfies 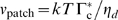. Ultimately the system eqs. 5, 6 and 7 will yield density profiles and a patch velocity 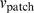 as a function of SNARE density 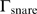.

## Results

### Exact Scaling Results for Patch Growth Velocity 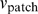


In this subsection we use scaling analysis to solve eqs. 4–7 for the steady state patch velocity 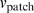. Results are presented first, followed by a brief summary of the analysis. A more detailed analysis is presented in Supplementary Material S1. Depending on the SNARE density 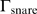, we find patch growth belongs to one of three regimes: fast, intermediate or slow. Our scaling results are exact deep within each regime where 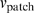 depends on 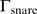 with a regime-specific power law:
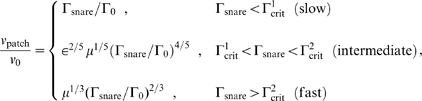
(8)where the diffusivities and complexation rate constant enter only through the dimensionless combinations
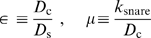
(9)and the characteristic scales for SNARE density and patch velocity are
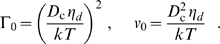
(10)


The regime boundaries are defined by two critical densities
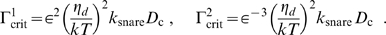
(11)


Note the intermediate regime exists because we assume SNARE diffusivity exceeds that of the complex (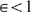). In subsequent subsections exact numerical solutions are presented which validate these scaling predictions. Realistic parameter values will then be used to obtain quantitative patch velocity predictions. We estimate that typical experimental densities belong to the fast regime, 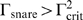 (see Parameter Values subsection).

### Derivation of Scaling Results

In this subsection we use our model to derive the results for patch velocity of eqs. 8–11. We find the velocity has power law dependence on SNARE density, with a different power in each of 3 regimes. The calculations below use scaling analysis. Later exact numerical solutions of the model equations (eqs. 4–7) will be presented which confirm the scaling results.

**Figure 3:**
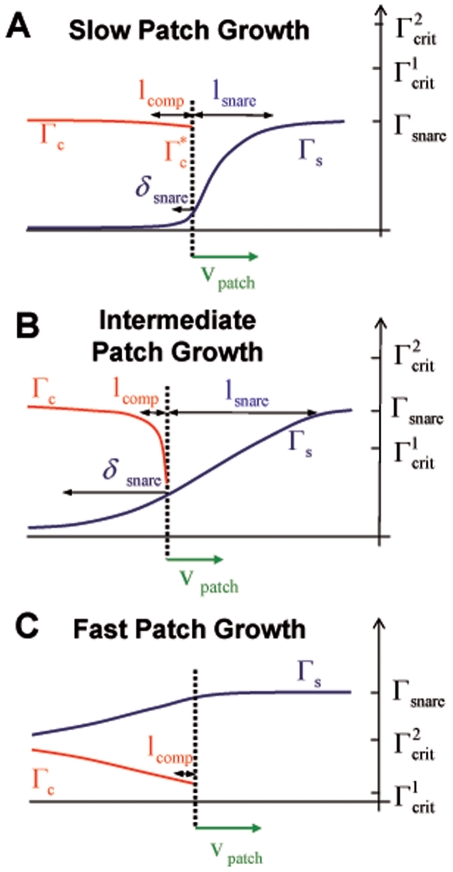
Schematic of steady state SNARE density profiles. Dashed line represents patch boundary moving with velocity 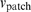, with patch to left of boundary. Uncomplexed SNAREs (density profile 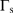, *blue*) bind within patch forming complexes (density profile 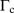, *red*). Far outside (inside) patch 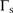 (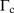) approaches the initial SNARE density, 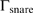. The vertical axis indicates the density scale and shows how 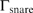 compares to two constant density values, 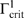 and 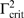. (A) Slow patch growth (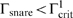). SNAREs bind rapidly inside the boundary and a diffusion-depleted zone of SNAREs develops outside the patch. The SNARE profile penetrates a small distance 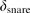 into the patch. Because the boundary moves slowly compared to complex diffusion the complex diffusion length 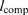 exceeds 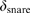 so 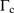 is relatively flat and the boundary density is undepleted, 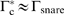. (B) Intermediate patch growth (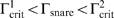.) As for the slow regime the SNARE profile is diffusion-depleted near the boundary. However patch growth is now fast relative to complex diffusion such that 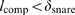; only a portion of those complexes generated in the patch catch up with the boundary before it moves on and the boundary density is depleted, 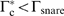. (C) Fast patch growth (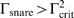). SNARE binding is slow compared to patch growth so SNARE density is little depleted at the boundary and 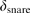 is large. As for the intermediate regime, 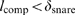 but the complex boundary density is even more depleted relative to 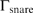.

Initially both adhering vesicles have uniform SNARE density, 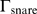. Subsequently SNARE complexation grows a patch. The SNARE complex density 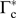 at the patch boundary drives patch growth, 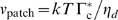 (eqs. 4, 6). In steady state the complex density deep inside the patch must equal 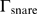 by number conservation, but depletion of complex density may occur near the boundary, 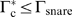. The extent of depletion and thus patch velocity depend on which regime a system belongs to (slow, intermediate or fast), which is determined by the SNARE-density-dependent ordering of three key length scales illustrated in Fig. 3. The first two scales are the diffusion lengths for uncomplexed SNARES 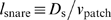 and for complexes 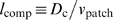. On length scales smaller than a given diffusion scale, diffusion is much faster than coherent patch boundary motion at velocity 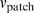. That is, the diffusive relaxation of the density profile on smaller scales than the corresponding diffusion length is so rapid that in effect the patch boundary is stationary during the relaxation episode. Note 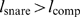. The third scale is the penetration depth 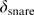 of the SNARE density profile into the patch. This is the typical separation between a SNARE's location and the patch boundary at the instant when it it complexes with a cognate SNARE, determined both by its own diffusion and the boundary movement. Another key quantity is the density of uncomplexed SNARES at the patch boundary, 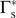, which may be depleted relative to the initial SNARE density, 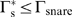.

**Slow regime,** 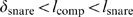 (Fig. 3A). We define this regime to be that where these three length scales are thus ordered. We will now show that this ordering is only true provided the SNARE density 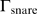 is less than a certain value, 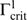. Now SNARE complexes are generated within the length 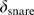 of the patch boundary. Since this is within the complex diffusion length 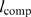 of the boundary, these newly created complexes are well mixed by diffusion so 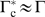 is undepleted. This immediately gives the slow regime patch velocity result of eq. 8, 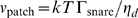. This regime is valid for small velocities where 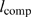 is larger than the SNARE penetration depth 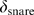. To determine this latter scale, note that the survival time 
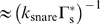
 of an uncomplexed SNARE entering the patch is determined by the SNARE density at the boundary 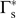. Since 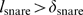 its displacement relative to the boundary during this period is dominated by its own diffusion, i. e. it penetrates the patch a distance 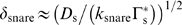. A second relation results from equating the rate of increase in the number of complexes in the patch to the complex production rate: 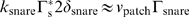. Eliminating 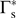 from these two relations one finds that 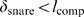 is only true if 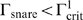 where 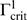 is given by the expression of eq. 11. It follows that this value of the SNARE density defines the upper limit of the slow regime.

**Fast regime,** 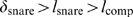 (Fig. 3C). Since the SNARE penetration depth is larger than the SNARE diffusion length, 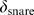 is determined by coherent patch motion rather than diffusion. After entering the patch SNAREs are left behind a distance 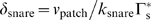 by the boundary in their survival time 
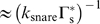
. Another consequence of 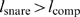 is that SNAREs are almost undepleted at the boundary, 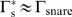. However since 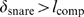, SNARE complex diffusion is inefficient over the region of complex production and complexes are depleted over the entire penetration length. Thus there is a hole in the complex density profile with slope 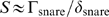. Due to diffusive mixing the complex boundary density is approximately equal to the average density over the region within the diffusion length 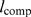 of the boundary, 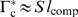. Using this in 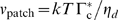 leads to the patch velocity expression of eq. 8 for the fast regime, 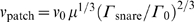. Self-consistency (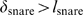) then leads to the requirement 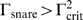 with 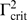 given by eq. 11.

**Intermediate regime,** 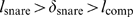 (Fig. 3B). This regime pertains for intermediate values of the SNARE density, 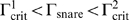. This corresponds to the situation where both interfacial densities 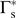 and 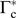 are depleted. The resulting power law 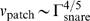 has exponent 4/5 lying between the 2/3 and 1 values for the fast and slow regimes, respectively. The reader is referred to the Supplementary Material S1 for the derivation.

### Parameter Values

In the following subsection the scaling solutions will be evaluated using realistic parameter values taken from or inferred from the literature, listed in table 1. The model equations will be numerically solved using these values and compared to the scaling predictions. This subsection describes how we are led to the values in Table 1.

**Table 1:** Parameter values for SNARE-mediated adhesion kinetics model.

Symbol	Meaning	Value	Source
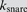	SNARE binding rate constant	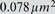 /s	[33], [42] 
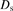	Uncomplexed SNARE diffusivity	2.6 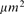 /s	[23] 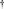
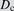	SNARE complex diffusivity	1.3 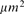 /s	[23] 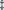
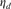	Patch growth drag coefficient	10^−3^ Pa s	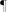
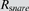	SNARE complexation range	8 nm	[33]
*d*	Initial membrane separation	3–61 nm	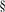
*γ*	GUV membrane tension	10^−6^–10^−2^ N/m	[35] 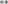

(*)Estimated using kinetic data for solubilized SNARE complexation [42] and 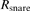 from ref. [33].

(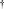)Average of syntaxin and synaptobrevin diffusivities in GUVs measured in [23].

(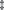)We estimate 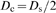.

(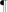)For *in vitro* experiments we take 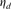 equal to the viscosity of water.

(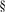)Minimum separation set by hydration forces [53]. Upper bound calculated from eq. 1 for lowest reported controllable GUV tension [35].

(**)Range of controllable tensions from ref. [35]. Upper bound corresponds to rupture tension.

#### Directly controllable parameters: tension, intermembrane separation and SNARE density

(i) Micropipette control allows direct regulation of GUV tension over a large range of values, shown in Table 1. (ii) Thus the initial separation between membranes in the GUV-GUV or GUV-SBL contact zone before patch nucleation is an experimentally variable parameter, being determined by tension and applied pressure. Ref. [36] describes a heavy-GUV-substrate contact zone where the pressure 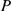 due to gravity is related to membrane tension and the GUV radius 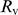 by 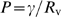, a relation which holds also if the pressure is applied by micropipette force. We used this relation together with the relationship between pressure, tension, and separation of eq. 1 to give the *d* values of Table 1. From the results of ref. [39] with van der Waals interactions set to zero we calculated the tension- and pressure-dependent prefactor *C* in eq. 1. We found 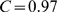 for the lower bound tension of Table 1; this increases five-fold over the range of tensions. Note that at physiological salt concentrations the range where van der Waals forces are strong is 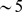 nm while hydration and electrostatic forces are even shorter range [37], [38]. Thus for almost all of the calculated separations in Table 1, 3 nm
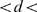
61 nm such that entropic repulsions dominate, justifying our use of eq. 1. Note that for most experimental tension values the membrane separation exceeds the SNARE complexation range, as assumed by our model. (iii) Patch growth is driven by SNARE density 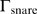 in the contacting membrane surfaces. We estimate the maximum attainable value is the density 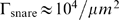 where sizable defects were observed for t-SNARE-reconstituted SBLs [10]. Below a certain level non-specific adhesion effects may swamp SNARE adhesion. As a practical lower bound we take the value 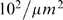 used in ref. [10], [12], among the lowest reported *in vitro* values. The range represents a two-decade SNARE density window to test GUV adhesion kinetics. For comparison, in cells the SNARE density presumably depends on organelle or vesicle type. A report of synaptic vesicle composition suggests 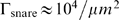 [43].

#### SNARE diffusivities

Bacia et al report values 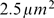/s and 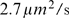/s for diffusivities of, respectively, syntaxin and synaptobrevin in GUVs [23] while 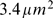/s was measured in ref. [24] for synaptobrevin in GUVs. In SBLs the value 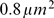/s was measured for t-SNAREs [44]. For our model calculations we take equal v- and t-SNARE diffusivities equal to a representative value 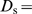
2.6 m^2^/*s*.

#### SNARE complex diffusivity

A key parameter is the diffusivity 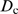 of a SNARE complex pinning two membranes together. We are not aware of measurements of this quantity. However, SNARE-adhered LUVs were mobile on GUV surfaces [24] suggesting SNARE complexes are mobile. For our model we adopt the simplest picture where the drag coefficient of a complex is the sum of the coefficients of the 2 SNAREs comprising the complex; the Einstein relation then implies 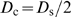 for uncomplexed SNAREs with equal diffusivities. Now because a SNARE complex pins two the membrane surfaces at a point (Fig. 1C) the diffusing complex must drag with it a double cone-like membrane structure (Fig. 1C). It is possible this may considerably increase its total drag coefficient and reduce 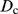 from our simple estimate above.

#### SNARE reach and 2D SNARE binding rate constant

The rate constant 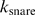 describes SNARE complexation in the 2D membrane world and has not been directly measured, to the best of our knowledge. However the 3D bulk rate constant for solubilized SNAREs was reported in ref. [42], 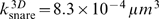/s. Since this value is far below the diffusion-controlled limit, it can be expressed 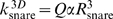 where 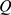 is the conditional binding rate *given* overlap of two cognate SNAREs and the SNARE reach 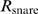 is analogous to the capture radius concept for chemical reactions. Assuming the local rate 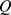 is unchanged in the membrane, one can similarly write 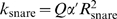. Taking, respectively, spherical and circular “reaction” regions the prefactors are 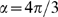, 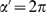. Thus 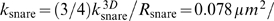
s after using 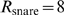
nm for SNARE reach. The latter is based on ref. [33] where forces in SNARE-reconstituted mica-supported lipid bilayers were measured with the surface force apparatus and SNAREs first interacted at membrane separation 8 nm.

**Patch drag coefficient,** 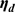 **.** The model of ref. [32] concluded that drag forces opposing patch growth are primarily due to hydrodynamic dissipation at the patch edge as intermembrane water is expelled, and 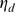 equals the viscosity of water multiplied by a geometric factor related to the angle at the membrane wedge just outside the patch. For simplicity we assume the geometric factor is close to unity. Thus we estimate the the drag coefficient equals the viscosity of water, 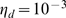 Pa s.

### Numerical Results and Confirmation of Scaling Laws

Using the parameter values of Table 1, in this subsection we obtain exact numerical solutions of the model describing SNARE-mediated adhesion, eqs. 4–7. The solution method is outlined in the Supplementary Material S1. The numerical solutions are compared to the analytical scaling results evaluated using the same parameter values. Numerical data and scaling predictions are in very close agreement.

#### Results for Table 1 parameters

**Figure 4:**
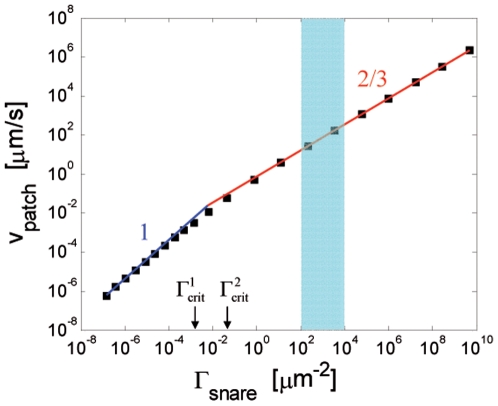
Model predictions for patch growth velocity as a function of SNARE density. Squares: exact numerical solutions of adhesion model using linear drag law (eq. 3 ) and table 1 parameters (corresponding dimensionless parameters: 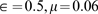). Solid lines show power law predictions of scaling analysis (eq. 8) for slow ( *blue*) and fast ( *red*) growth regimes with indicated exponents. The numerical results confirm the asymptotic power law predictions from scaling analysis. Note the intermediate regime is non-existent since critical densities (shown) are not well separated. Error in the scaling predictions (relative to the numerical values) is maximum at the crossover from slow to fast regimes (130%), and approaches zero far from the critical densities. Shaded area represents accessible range of 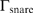 values *in vitro* (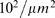 to 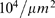) which are deep in the fast regime. The v-SNARE density of synaptic vesicles (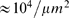 [43]) suggests *in vivo* 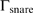 values may lie in this range.

Figure 4 presents numerical results for patch velocity versus SNARE density. Table 1 parameters correspond to 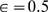, 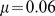. Plotted for comparison are the power law analytical results of eq. 8. The agreement with the scaling predictions is excellent in the slow and fast regimes: the power laws with exponents 1 and 2/3 are unambiguously confirmed. The results indicate the intermediate regime is “squeezed out” for these parameter values, as expected since the diffusivity ratio 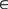 is close to 1.

The shaded blue window indicates the estimated practically accessible *in vitro* SNARE density range (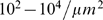). This lies deep in the fast regime, i. e. well to the right of the upper critical density 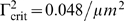 (eq. 11). The corresponding predicted patch velocities lie in the range 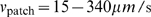/s which is in principle readily measurable using optical imaging. Velocities are high near the upper bound density because the SNAREs are dense and many are available to complex and provide osmotic pressure. At this maximum practical density, SNAREs are nearly shoulder-to-shoulder if one takes the maximum packing density to be 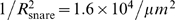.

#### Results for other parameter values: the universal velocity-density relation

**Figure 5:**
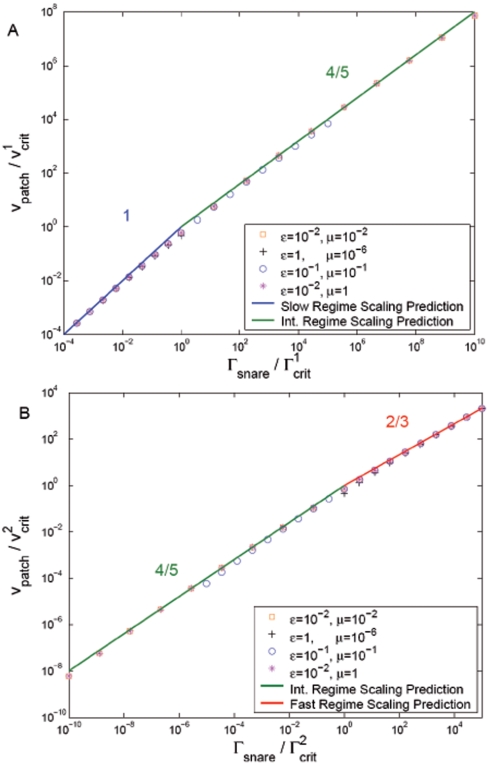
Collapse of scaled SNARE-mediated adhesion data onto universal patch growth laws. Symbols indicate exact numerical solutions of patch growth model using the linear drag law (eq. 3 ). SNARE parameters are varied through the dimensionless combinations 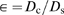 and 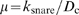 as shown. Solid lines indicate scaling analysis power law solutions (eq. 8 ) for slow ( *blue*), intermediate ( *green*), and fast ( *red*) growth regimes with indicated exponents. (A) Patch velocity versus SNARE density in slow and intermediate regimes. Density scaled by 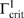 and velocity scaled by the corresponding patch velocity 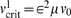. The right-most point of each data set corresponds to 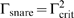. (B) As for (A), but for intermediate and fast regimes. Densities and velocities scaled, respectively, by 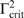 and 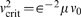. The left-most point of each data set corresponds to 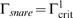; note the width of the intermediate regime is larger for smaller values of 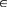. Numerical results confirm the asymptotic solutions with relative errors in velocity peaking at the critical densities (70% in (A) and 44% in (B)) and approaching zero far from the critical densities in each regime.

In Fig. 5 patch velocity predictions are presented for parameter values outside those of Table 1. This is important both because of uncertainty in some parameters, and because the values will presumably depend on the type of SNARE. Now in the model predictions for patch velocity the SNARE diffusivities and complexation rate constant enter only through the dimensionless combinations 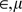. Figure 5A shows numerically calculated velocities versus SNARE density in the slow and intermediate regimes for a range of SNARE parameter values varied through 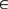 and 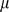. Figure 5B shows the same for the intermediate and fast regimes. SNARE densities were scaled with the critical values (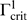 or 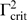) and patch growth velocities with the corresponding velocities at the regime boundaries (named 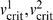 in Fig. 5).

Figure 5 leads to two important conclusions. (1) When SNARE density and patch velocity are scaled as above, the velocity-density relationship *collapses* onto a single universal curve. In other words, all dependence on the parameters characterizing the SNARES – diffusivities and complexation rate constant – appears only in the critical densities and velocities. (2) The universal curve onto which the numerically obtained data collapses is in very close agreement with our earlier scaling predictions, eq. 8. The predicted power laws in each regime are clearly obeyed.

### Fusion Kinetics

Though adhesion is our main concern in this paper, we briefly consider a very simple model of fusion whose results articulate how adhesion and fusion may be coupled. Past theoretical work on fusion has focused mainly on protein-free membranes. Energy barriers to access intermediate high-curvature membrane structures on the pathway to fusion were calculated [45], [46]. It has been proposed that similar lipidic structures may be realized in protein-mediated fusion [3], [47]. We are not aware of first principles models quantitatively predicting the kinetics of SNARE-mediated fusion, based on a microscopic picture from the SNAREs upward.

Here we invoke the simplest imaginable model for SNARE-induced GUV fusion: each SNARE complex in the patch can trigger fusion with a certain probability per unit time, 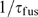, *independently* of all others. Only one such event can occur, assuming fusion results in immediate and irreversible conversion of the adhered vesicles into a single vesicle.

What is the delay before fusion occurs? This depends on the mean fusion time 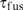 for a single SNARE complex, but also on how rapidly the total number of SNARE complexes in the patch increases with time; the more SNAREs, the higher the fusion probability per unit time. Thus fusion kinetics depend on the adhesion kinetics we have analyzed.

#### Calculation of distribution of fusion times

Adhesion kinetics analyzed in previous subsections determine the number of SNARE complexes 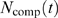 created in the patch after time *t*. For constant velocity 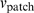,
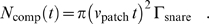
(12)


Assuming each SNARE complex triggers fusion independently from all others in the mean time 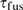, the (“survival”) probability that no fusion has occurred after time *t* is
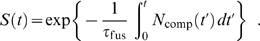
(13)


This is the product of factors 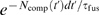, namely the probability no fusion occurs in the interval 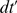 given fusion probability per unit time 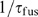.

The distribution of fusion times is thus
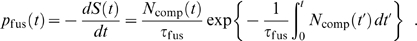
(14)


Inserting the particular form 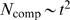 of eq. 12 gives
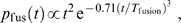
(15)where the mean fusion time is
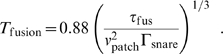
(16)


**Figure 6:**
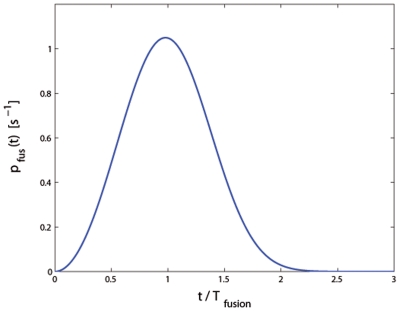
Predicted distribution of fusion times in the many-SNARE fusion regime (eq. 15 ). The distribution of fusion times is very broad, 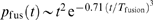, characterized by the mean fusion time 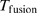 determined by SNARE density. Time is measured from the instant of adhesion patch nucleation.

This distribution of fusion times 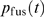 is very broad (see Fig. 6). It follows that 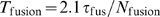 where 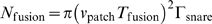 is the number of complexes assembled in the patch by the mean fusion time. This quantifies how fusion is accelerated when many SNAREs act in parallel.

#### Prediction for mean vesicle fusion time

The mean vesicle fusion time given by eq. 16 depends on the SNARE density and patch velocity. Thus we predict three regimes of fusion kinetics depending on SNARE density corresponding to the three regimes of adhesion kinetics. The dependence of the mean vesicle fusion time on SNARE density is obtained by combining the predictions for patch velocity of eq. 8 for each regime with eq. 16:
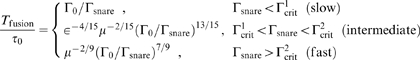
(17)where a new timescale appears, 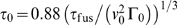. Note that for the parameters of Table 1 adhesion and fusion kinetics lie deep in the fast regime where 
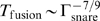
. Thus as density is increased from the minimum to maximum values of the accessible *in vitro* range (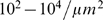) the mean fusion time is predicted to be reduced by a factor of 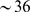. This strong dependence of 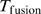 on SNARE density is because increasing 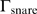 speeds up fusion by increasing the number of SNARE complexes in the patch in two ways: (i) increasing the patch growth rate (eq. 8 ), and (ii) increasing the SNARE complex density inside the patch.

The above analysis implicitly assumed that before the fusion event sufficient time had elapsed that a steady state adhesion patch had been established (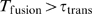) containing many complexes (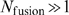 or 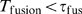). Here 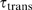 is the duration of the transient growth regime following nucleation at 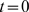 but preceding steady state. Thus eq. 17 is self-consistent provided
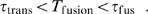
(18)


The second inequality can be restated as
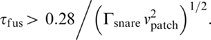
(19)


In the Discussion section we argue that these inequalities are satisfied for some *in vitro* systems.

#### Single SNARE fusion

Finally, if the single SNARE fusion time 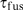 is so small that after patch nucleation a second complex had insufficient time to develop then fusion is triggered by a single SNARE. In this case 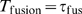 and fusion times follow a simple Poisson distribution, 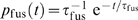. Now the complex production rate just after patch nucleation by the very first complex is 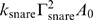 where 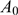 is the area surrounding the first complex where cognate SNAREs can reach one other. From Helfrich theory [38] we estimate 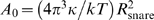 where 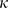 is the membrane bending modulus. Thus the condition for single SNARE fusion kinetics is estimated as
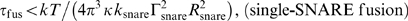
(20)


## Discussion

In this paper we modeled interactions between two large SNARE-reconstituted membranes as in GUV-GUV or GUV-SBL experiments. Such experiments are relatively unexplored but may provide unique information unavailable from the widely exploited SUV-based methods.We predict SNARE complexation creates an adhesion patch whose growth rate is determined by SNARE density.

### Predictions of model: power law increase of adhesion patch growth rate with SNARE density

Using parameter values inferred from available experimental data (see Table 1) results are shown in Fig. 4. Patch growth velocity is driven by the initial SNARE surface density and grows as a power law, 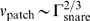, a directly testable prediction. Predicted patch growth speeds are 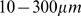/s for typical *in vitro* SNARE densities (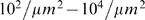).

### Three patch velocity regimes

At low SNARE densities (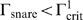) we found uniform complex density in the patch and 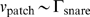 is independent of SNARE complexation rate constant 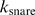 (slow regime). This was identified in ref. [32]. At intermediate (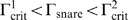) and high (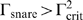) densities 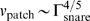 and 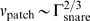, respectively (intermediate and fast regimes). Increased growth rate now outstrips diffusion of complexes whose density at the patch boundary is thus depleted resulting in progressively weaker power laws and growth rates depending on 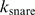 and diffusivities. These predictions were confirmed by numerical solutions (Figs. 4,5). For the SNARE parameters of Table 1 typical SNARE densities belong to the fast regime. Note the exponent 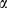 in the growth law 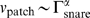 decreases with increasing SNARE density. This is a general trend and does not require a linear drag law as we assumed to obtain the above results. In the Supplementary Material S1 we treat the alternative (non-linear) drag law proposed in ref. [32], 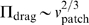. We find qualitatively unchanged behavior, but growth exponents are modified to 3/2, 1, and 3/4 in the slow, intermediate, and fast regimes, respectively (see Supplementary Figure S1).

### Prediction of constant patch growth velocity

We found that our model equations describing SNARE density profile evolution and patch growth (eqs. 1–3 of the Supplementary Material S1) have long time solutions where the patch grows at constant velocity, 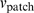. The solutions obey eqs. 5–7 in the main text. We stress that constant patch velocity is not an assumption of our model but emerges from the governing equations. The following stability argument helps to physically motivate why patch growth speed settles down to a constant value 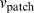. Were patch growth to momentarily diminish from this value, say, additional time would be available for SNARE complexes to assemble in the patch and diffuse to the boundary, boosting the complex boundary density and osmotic pressure and tending to restore the velocity to its former higher level. On the other hand a sudden increase in velocity relative to the steady state value would deplete the complex boundary density and tend to drive the patch velocity down again.

### Transient preceding constant patch growth

The constant patch growth regime and the main results presented here are valid at times sufficiently large that patch size exceeds the size of the region close to the boundary where SNARE and complex density profiles change. In the fast regime the requirement is that patch size exceeds the SNARE penetration depth 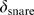 which is the size of the depletion region in the complex density profile within the patch. For the practically accessible SNARE density range indicated in fig. 4 this scale ranges from 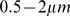.

### Other parameter values

Understanding adhesion kinetics for parameter values besides those in Table 1 is important. (i) Different SNARE types will presumably have different parameter sets. (ii) Future GUV studies may enable more confident inference of parameter values such as 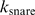 by fitting model predictions to experiment. (iii) Some parameters may be experimentally manipulated, e.g. by using recombinant SNAREs with modifications or drugs such as toxins which cleave SNAREs at specific sites. Physical properties could be adjusted (e.g. the drag coefficient 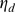 by high viscosity additives). Scaling analysis showed that patch kinetics depend on parameters through the combinations 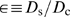 and 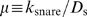 only. An important prediction from scaling analysis, confirmed numerically, is that patch growth versus SNARE density collapses onto universal curves for different parameter values (Fig. 5).

### Validity of 2D SNARE complexation kinetics

In the tightly adhered patch region we assumed apposing membranes were so close that SNAREs are in reach of each other when laterally aligned. This is a reasonable assumption since already formed SNARE complexes themselves are the agents holding the membranes together. Thus a complexation event within a developed patch does not require local bending of the membranes toward one another to bring cognate SNAREs together. Hence complexation kinetics are effectively 2D, with 2D SNARE binding rate constant 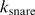. This assumption is valid provided the mean membrane undulation amplitude 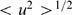 is less than the SNARE reach 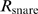. Applying the Helfrich formula 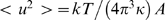 for a membrane patch of typical bending modulus 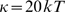 [35] and area 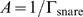 (the mean area between complexes in the patch since 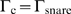 away from boundary) yields the necessary condition 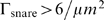. This is easily satisfied for typical experimental SNARE densities (see Fig. 4). For the lowest densities, some corrections may be expected because complex density may be reduced near the patch boundary.

### Fusion kinetics are coupled to adhesion kinetics

Implementing the simplest assumption that complexes promote fusion independently, we found a broad distribution of fusion times 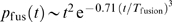 where the mean fusion time 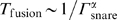 decreases at higher SNARE density with a regime-dependent exponent 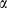. Generally, if fusion is slow enough that many complexes can first assemble the fusion probability per unit time should *increase* due to adhesion kinetics. A second possibility is that the individual SNARE complex fusion time 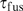 is so small that fusion would occur almost instantly on production of the first complex. Fusion times would then follow the much less broad exponential one-SNARE distribution, 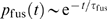.

### Estimating mean fusion time

Fusion time predictions from our simple fusion model, eq. 17, are self-consistent provided the conditions of eqs. 18, 19 are satisfied. Taking density 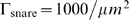 with table 1 parameters gives 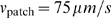 (eq. 8, fast regime) so the requirement of eq. 19 is that the single SNARE complex fusion time 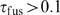 ms. We estimate the transient duration as the time for the patch size to grow larger than the depleted region at the boundary of size 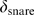, 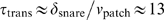 ms. Thus 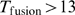 ms is also required. Now the predicted mean GUV fusion time 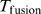 (eq. 16 ) depends on 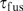 which has not been measured. In SUV experiments due to geometric constraints each SUV may be docked and fused by order one SNARE complexes. Thus measured SUV fusion times may provide a crude estimate of 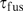. (i) Using the value 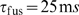 from ref. [10], eq. 16 gives 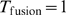 ms which belongs to the transient regime. Thus our predicted fusion time is inaccurate but we can conclude fusion occurs in the transient many-SNARE regime. (ii) Using instead 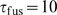 min from refs. [3], [9], eq. 16 gives 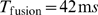. This value satisfies the self-consistency conditions of eq. 18. (iii) With the intermediate value 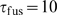 s from refs. [11], [12], eq. 16 gives 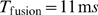, close to the transient-steady state boundary. This value is approximately self-consistent and provides at least a crude estimate. Note that the single SNARE fusion condition of eq. 20 reads 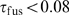 ms; thus all cases (i)-(iii) are in many-SNARE regimes.

### Patch nucleation

Important issues not addressed here are patch nucleation times and whether additional patches can nucleate before fusion. The probability a second patch nucleates within the fusion time 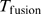 is 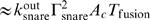, where 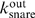 is the reduced binding constant outside the patch. Estimating the initial vesicle-vesicle contact area 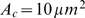 and using Table 1 parameters with 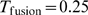 s, the condition 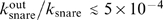 must be satisfied to ensure only one patch develops. An interesting experimental possibility would be to tune 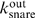 by reducing (increasing) membrane tension to increase (reduce) the membrane separation according to eq. 1.

### Implications for cellular fusion pathways

**Figure 7:**
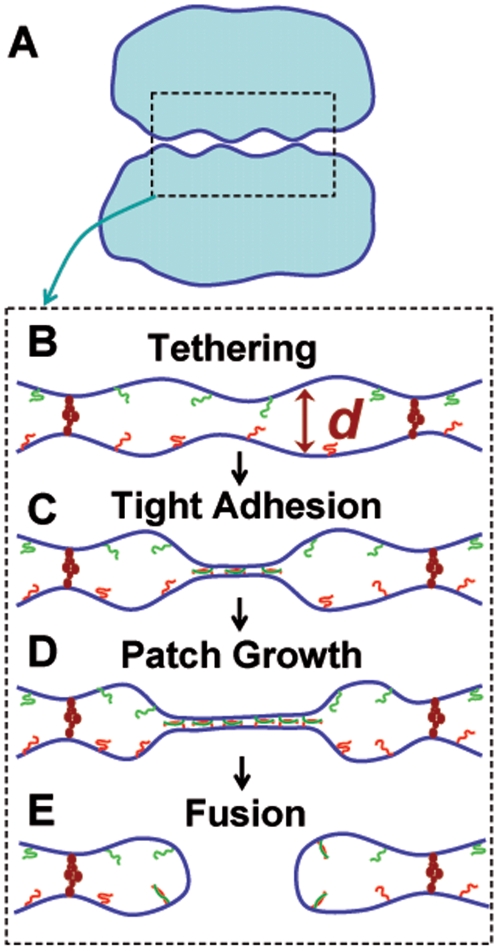
Tethering factors and possible fusion sequence for large compartments in vivo. (A) Large micron-scale compartments loosely bound by tethering factors which are thought to mediate the first membrane contact on the fusion pathway. (B–E) Blow-up of boxed region in (A), showing possible sequence from tethering to fusion. (B) Tethering factors ( *brown*) loosely bind the compartments in preparation for SNARE action, setting the initial membrane separation *d*. If tether size (∼30 nm) sets 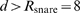 nm, binding of t-SNAREs ( *red*) and v-SNAREs ( *green*) is hindered. (C) After patch nucleation by the first SNARE complex, aided by direct SNARE-tether interactions or membrane fluctuations, complex assembly and patch growth is facilitated by the tightly adhered patch where SNAREs are in reach. SNARE assembly may be regulated and organized by additional factors such as SM proteins (not shown). (D) As more complexes develop, the self-promoting adhesion patch grows, possibly driven by SNARE complex osmotic pressure. (E) Fusion is triggered within the patch by SNAREs individually or as part of a multi-component fusion machine.

A possible pathway to cellular fusion is depicted in Fig. 7. Biological membrane tensions *γ* may be sufficiently low that Helfrich repulsions work against adhesion. Using 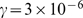 N/m (measured from plasma membrane blebs lacking cytoskeletal adhesion [48]) to estimate the tension of large cellular compartments, eq. 1 predicts membrane approach closer than 37 nm is strongly suppressed (eq. 1). Tethering factor sizes (∼30 nm [34]) suggest they may reach across this gap to loosely bind membranes before SNARE-mediated adhesion [49], [50], as illustrated in Fig. 7B. The transition of secretory granules between tethered and firmly docked states involved a 20-nm step toward the plasma membrane which presumably corresponded to SNARE adhesion [51]. Thus tethers may establish a contact zone where mean membrane separation exceeds SNARE reach, ready for subsequent complexation events to grow a tight SNARE adhesion patch (Fig. 7A–D) in readiness for fusion (Fig. 7E). An important quantitative difference compared to *in vitro* is that frictional resistance to patch growth may be much higher *in vivo* since estimates of cytoplasmic viscosity [52] range from 1 to 10^7^ that of water.

## Supporting Information

Supplementary Materials S1 (0.10 MB PDF)

Figure S1 Collapse of scaled SNARE-mediated adhesion data onto a single universal patch growth law. Same as Fig. 5 of the main text, but using non-linear relation between velocity and patch boundary complex density, eq. S19. Symbols indicate exact numerical solutions of patch growth model for a range of parameter values as shown. Solid lines denote scaling predictions. (A) Patch velocity versus SNARE density in slow and intermediate regimes. Density scaled by Γ^1^_crit_ and velocity scaled by v^1^_crit_ = ε^3/2^λ^3/4^v_0_. (B) As for (A), but for intermediate and fast regimes. Densities and velocities scaled, respectively, by Γ^2^_crit_ and v^2^_crit_ = ε^-3/2^λ^3/4^v_0_. Numerical results confirm the asymptotic solutions with relative errors in the velocity peaking at the critical densities (106% in (A) and 54% in (B)) and approaching zero far from the critical densities in each regime.
(0.64 MB TIF)
