# Supplementary material for: Model of SNARE-Mediated Membrane Adhesion Kinetics
Source: PLoS One. 2009 Aug 3;4(8):e6375. doi: 10.1371/journal.pone.0006375 (PMC2715897; doi:10.1371/journal.pone.0006375)
Supplement: Supplementary Materials S1 — (0.10 MB PDF) [file pone.0006375.s001.pdf]

Model of SNARE-Mediated Membrane Adhesion Kinetics: Supplementary Material S1

Jason M. Warner[1], Erdem Karatekin[2], Ben O'Shaughnessy[3]
1 Jason M. Warner, Department of Chemical Engineering, Columbia University, New York, NY, USA
2 Erdem Karatekin, Institut de Biologie Physico-Chimique, Centre National de la Recherche Scientifique UPR 1929, Paris, France
3 Ben O'Shaughnessy, Department of Chemical Engineering, Columbia University, New York, NY, USA

Equations Governing SNARE Density Profiles

Let $\widetilde{\Gamma}_{\rm s}(r,t)$ and $\widetilde{\Gamma}_{\rm c}(r,t)$ denote the density of SNAREs and complexes, respectively, a distance r from the patch center. Note $\widetilde{\Gamma}_{\rm c}(r,t) = 0$ for $r > R_{\rm p}(t)$. The evolution equations read

$$\frac{\partial \widetilde{\Gamma}_{\rm s}}{\partial t} = D_{\rm s}\,\nabla_r^2\,\widetilde{\Gamma}_{\rm s} - \; k(r)\,\widetilde{\Gamma}_{\rm s}^2\;, \quad \frac{\partial \widetilde{\Gamma}_{\rm c}}{\partial t} = D_{\rm c}\,\nabla_r^2\,\widetilde{\Gamma}_{\rm c} + \; k(r)\,\widetilde{\Gamma}_{\rm s}^2\;, \tag{S1}$$

where the binding rate constant is only non-zero within the patch,

$$k(r \le R_{\rm p}(t)) = k_{\rm snare}\;, \quad k(r > R_{\rm p}(t)) = 0\;, \quad \frac{dR_{\rm p}}{dt} = \frac{kT\Gamma_{\rm c}^*}{\eta_d}\;, \quad \Gamma_{\rm c}^* \equiv \widetilde{\Gamma}_{\rm c}(R_{\rm p},t) \quad . \tag{S2}$$

Boundary conditions are: (i) zero SNARE complex flux across the moving patch boundary and (ii) the far-field density (at $r = \infty$ for large vesicles relative to patch size) is the initial SNARE density:

$$\left[\nabla_r \widetilde{\Gamma}_{\rm c}\right]_{r=R_{\rm p}(t)} = -\frac{kT\Gamma_{\rm c}^{*2}}{\eta_d D_{\rm c}}\;, \qquad \widetilde{\Gamma}_{\rm s}(\infty,t) = \Gamma_{\rm snare} \quad . \tag{S3}$$

After a transient a steady state patch growth velocity $v_{\rm patch}$ results. For patch radius $R_{\rm p}(t)$ much larger than the size of the region near the patch boundary where SNARE densities change substantially, the geometry becomes approximately 1D orthogonal to the patch boundary (x direction) and the far field boundary conditions are in effect at $x = \pm\infty$. In steady state the density fields are unchanging in a frame of reference moving with the boundary. This density field for the SNAREs is named $\Gamma_{\rm s}(x) \equiv \widetilde{\Gamma}_{\rm s}(r,t)$ where $x \equiv r - v_{\rm patch}t$ and $\Gamma_{\rm c}(x)$ for the complexes is defined similarly. Thus the general equations S1, S2, and S3 become the steady state equations 5, 6, and 7 in the main text, respectively. In eq. 7, the complex boundary condition deep inside the patch ("$x = -\infty$") follows from SNARE number conservation.

Alternative Time Scale-Based Derivation of Scaling Results, Eqs. 8 and 11

In Exact Scaling Results of the main text a brief derivation of our scaling results for patch velocity in the slow and fast patch growth regimes was presented. More detailed derivations are given below, including derivation of the intermediate regime result. The arguments below are phrased in terms of key time scales, an alternative approach to the length-scale-based derivations in the main text.

Key time and length scales

The driving force for patch growth is the SNARE density in each vesicle, $\Gamma_{\rm snare}$. When this is far from the critical densities (eq. 11 of main text) the key timescales are well separated and $v_{\rm patch}$ obeys exact power laws as a function of $\Gamma_{\rm snare}$. If the patch has a definite growth velocity $v_{\rm patch}$, there are 3 characteristic timescales:

$$\tau_{\rm bind} = \frac{1}{k_{\rm snare}\Gamma_{\rm snare}}\;, \qquad \tau_{\rm D}^{\rm s} = \frac{D_{\rm s}}{v_{\rm patch}^2}\;, \qquad \tau_{\rm D}^{\rm c} = \frac{D_{\rm c}}{v_{\rm patch}^2} \quad . \tag{S4}$$

The first is the SNARE-SNARE binding time, the time required for all SNAREs at density $\Gamma_{\rm snare}$ in one vesicle to undergo binding "reactions" in the patch region with cognate SNAREs in the apposing vesicle. $\tau_{\rm D}^{\rm s}$ is the SNARE diffusion time when SNARE diffusive displacement and patch boundary displacement just match ($(D\tau_{\rm D}^{\rm s})^{1/2} = v_{\rm patch}\tau_{\rm D}^{\rm s}$); at longer times coherent patch motion beats diffusion. It can be thought of as the time a given SNARE initially near the interface remains close to it through its own diffusion before the interface moves onward. Similarly $\tau_{\rm D}^{\rm c}$ is the diffusion time of a SNARE-SNARE complex produced by a binding event. We will see the qualitative behavior depends on the relative magnitude of these scales.

Closely related to $\tau_{\rm D}^{\rm c}$ is the SNARE complex diffusion length,

$$l_{\rm comp} = v_{\rm patch}\tau_{\rm D}^{\rm c} \quad . \tag{S5}$$

The outward osmotic pressure benefits only from complexes produced within $l_{\rm comp}$ of the patch boundary.

Uncomplexed SNARE density profile in steady state

To self-consistently calculate the interfacial SNARE complex density setting velocity, the uncomplexed SNARE profile $\Gamma_{\rm s}(x)$ must first be determined. $\Gamma_{\rm s}(x)$ undergoes a transition from $\Gamma_{\rm snare}$ far from the patch to zero deep inside the patch (Fig. 3). Suppose $v_{\rm patch}$ is given (later, it will be determined self-consistently from eq. 4 of the main text once $\Gamma_{\rm c}^*$ has been calculated). What is the SNARE density $\Gamma_{\rm s}^*$ at the patch boundary?

Consider first a slowly growing patch, such that $\tau_{\rm bind} < \tau_{\rm D}^{\rm s}$: before the boundary has "moved on" there is sufficient time for all SNAREs inside the patch to undergo binding reactions. Thus a depletion hole in the SNARE profile $\Gamma_{\rm s}(x)$ develops outside the boundary and $\Gamma_{\rm s}$ is small inside the patch including the interfacial density, $\Gamma_{\rm s}^* \ll \Gamma_{\rm snare}$. The SNARE profile penetrates a small distance $\delta_{\rm snare}$ into the patch, equal to the distance SNAREs can diffuse before they bind after a time $\sim 1/k_{\rm snare}\Gamma_{\rm s}^*$,

$$\delta_{\rm snare} \approx (D_{\rm s}/k_{\rm snare}\Gamma_{\rm s}^*)^{1/2} \ , \qquad k_{\rm snare}\,\Gamma_{\rm s}^{*2}\,\delta_{\rm snare} = v_{\rm patch}\Gamma_{\rm snare} \quad . \tag{S6}$$

Note $\delta_{\rm snare}$ is determined by diffusion because on the binding timescale the boundary is effectively stationary since the diffusion time is much greater. The second relation above follows by number conservation: in steady state the rate SNAREs are consumed by binding reactions within the region of width $\sim \delta_{\rm snare}$ equals the rate new SNAREs are input as the patch grows. Solving eq. S6 for the interfacial density gives

$$\frac{\Gamma_{\rm s}^*}{\Gamma_{\rm snare}} \approx \begin{cases} (\tau_{\rm bind}/\tau_{\rm D}^{\rm s})^{1/3} \ , & \tau_{\rm bind} < \tau_{\rm D}^{\rm s} \\ 1 \ , & \tau_{\rm bind} > \tau_{\rm D}^{\rm s} \ . \end{cases} \tag{S7}$$

The second result is in the opposite limit of a rapidly growing patch, $\tau_{\rm bind} > \tau_{\rm D}^{\rm s}$. In this case when a SNARE first enters the patch the probability it will bind before the boundary moves on is very small: the density is undepleted at the boundary, $\Gamma_{\rm s}^* \approx \Gamma_{\rm snare}$, there is no diffusion hole outside the patch and within the patch the SNARE profile tails off relatively gradually (see Fig. 3).

Slower patch growth, $\tau_{\rm bind} < \tau_{\rm D}^{\rm s}$

Consider first SNARE densities low enough that the SNARE diffusion time exceeds the binding time. Then the SNARE interfacial density is small (see eq. 4, main text) as is the penetration depth of SNAREs into the patch, $\delta_{\rm snare}$ (Fig. 3*b*). The interfacial complex density is governed by the ratio of this scale to the complex diffusion length. Now from eqs. 4 and 11 of the main text and S5 and S6, this ratio obeys

$$\left(\frac{\delta_{\rm snare}}{l_{\rm comp}}\right)^3 = \frac{\tau_{\rm bind}}{\epsilon^2\tau_{\rm D}^{\rm c}} = \frac{\Gamma_{\rm snare}}{\Gamma_{\rm crit}^1} \quad . \tag{S8}$$

It follows that if the binding time is very small ($\tau_{\rm bind} < \epsilon^2 \tau_{\rm D}^{\rm c}$) or equivalently the SNARE density is less than a critical value $\Gamma^1_{\rm crit}$, complex production is effectively a delta function source at the boundary, $\delta_{\rm snare} < l_{\rm comp}$. All generated complexes can contact the boundary, $\Gamma_{\rm c}^* = \Gamma_{\rm snare}$. Thus $v_{\rm patch} = (kT/\eta_d)\Gamma_{\rm snare}$ is the linear relation shown in eq. 8 of the main text.

At higher densities the complex diffusion length is less than the SNARE penetration depth. Of complexes generated in a region of width $\delta_{\rm snare}$, a small fraction $l_{\rm comp}/\delta_{\rm snare}$ are within diffusive range of the interface and contribute to $\Gamma_{\rm c}^*$:

$$\frac{\Gamma_{\rm c}^*}{\Gamma_{\rm snare}} = \frac{l_{\rm comp}}{\delta_{\rm snare}} = \epsilon^{2/3}\left(\frac{\tau_{\rm D}^{\rm c}}{\tau_{\rm bind}}\right)^{1/3} , \qquad \Gamma^1_{\rm crit} < \Gamma_{\rm snare} < \Gamma^2_{\rm crit} \quad . \tag{S9}$$

Using eqs. 4 (main text) and S4, this gives a different power law relation, $v_{\rm patch}/v_0 = \epsilon^{2/5}\mu^{1/5}(\Gamma_{\rm snare}/\Gamma_0)^{4/5}$, as shown in eq. 8. This intermediate regime is valid up to the second critical density given by eq. 11, obtained by setting $\tau_{\rm bind} = \tau_{\rm D}^{\rm s}$ and using the velocity law in eq. 8 (main text) for the intermediate regime.

Fast patch growth, $\tau_{\rm bind} > \tau_{\rm D}^{\rm s}$

At high enough SNARE densities binding is relatively slow and the SNARE boundary density is undepleted, $\Gamma_{\rm s}^* \approx \Gamma_{\rm snare}$. The complex density at the boundary is the fraction of these SNAREs binding during the complex diffusion time

$$\Gamma_{\rm c}^* \approx \frac{\tau_{\rm D}^{\rm c}}{\tau_{\rm bind}}\Gamma_{\rm snare} , \qquad (\Gamma_{\rm snare} > \Gamma^2_{\rm crit}) \quad . \tag{S10}$$

After using eq. 4 of the main text and eq. S4, this gives $v_{\rm patch}/v_0 = \mu^{1/3}(\Gamma_{\rm snare}/\Gamma_0)^{2/3}$, the high density regime of eq. 8.

Numerical Solution Methods

The steady state SNARE-mediated adhesion and patch growth kinetics eqs. 5, 6 and 7 of the main text were solved numerically as follows. First, a helpful result is derived. Assume $v_{\rm patch}$ is given. Multiplying the differential equation for SNARE complexes (the 2nd of eq. 5) by $e^{(v_{\rm patch}/D_{\rm c})x}$ the equation is rewritten ($x < 0$)

$$\frac{d}{dx}\left(\frac{d\Gamma_{\rm c}}{dx}\, e^{(v_{\rm patch}/D_{\rm c})x}\right) = -\frac{k_{\rm snare}}{D_{\rm c}}\,\Gamma_{\rm s}^2\, e^{(v_{\rm patch}/D_{\rm c})x} \quad . \tag{S11}$$

Integrating, one has

$$\left[\frac{d\Gamma_{\rm c}}{dx}\right]_{x=0} = -\frac{k_{\rm snare}}{D_{\rm c}}\int_{-\infty}^{0} \Gamma_{\rm s}^2(x)\, e^{(v_{\rm patch}/D_{\rm c})x} dx \quad . \tag{S12}$$

Using the no-flux patch boundary condition in eq. 7 of the main text we obtain the following expression for the complex density at the patch boundary in terms of the uncomplexed SNARE profile $\Gamma_{\rm s}(x)$:

$$\Gamma_{\rm c}^* = \frac{k}{v_{\rm patch}}\int_{-\infty}^{0} \Gamma_{\rm s}^2(x)\, e^{(v_{\rm patch}/D_{\rm c})x}\, dx \quad . \tag{S13}$$

The power of this result is that the complex boundary density $\Gamma_{\rm c}^*$ can be obtained directly from the uncomplexed SNARE profile without the need to calculate the full complex profile $\Gamma_{\rm c}(x)$. Our numerical procedure is as follows.

Step 1. A patch velocity $v_{\rm patch}$ is assumed. The SNARE profile $\Gamma_{\rm s}(x)$ is then calculated from the differential equation governing uncomplexed SNAREs, eq. 5 of main text, using a 4th-order Runge Kutta method.

Step 2. The profile $\Gamma_s(x)$ then yields Γ_c^* from eq. S13.

Step 3. Γ_c^* is inserted into the drag law (eq. 4 of the main text for the linear drag law, or S14 for alternative law of next section) and solved for the patch velocity. We call this value $v'_{\rm patch}$.

Step 4. If $|v'_{\rm patch}/v_{\rm patch} - 1|$ is sufficiently small, $v_{\rm patch}$ is the self-consistent numerical solution. If not, a new improved value for $v_{\rm patch}$ is selected and steps 1-4 repeated.

Results for Alternative, Non-linear Patch Drag Law

All results of the main text for adhesion and fusion kinetics were obtained assuming the drag force resisting patch growth depends linearly on the patch velocity $v_{\rm patch}$. This led to a simple linear local relation between velocity and patch boundary complex density, $v_{\rm patch} \sim \Gamma_c^*$ (eq. 4). Here we present results using the non-linear local relation proposed by de Gennes et al [32],

$$v_{\rm patch} = \frac{\gamma}{\eta}\left(\frac{kT\,\Gamma_c^*}{\gamma}\right)^{3/2} \text{(alternative form)}\ , \tag{S14}$$

which involves the vesicle tension γ and the viscosity η of the intermembrane fluid.

In effect, this relation results from a *non-linear* drag law $\Pi_{\rm drag} = \gamma(v_{\rm patch}/\widetilde{v}_0)^{2/3}$, where $\widetilde{v}_0 = \gamma/\eta$, replacing eq. 3 of the main text. Balancing this with the SNARE complex osmotic pressure leads to the above result eq. S14. The authors of ref [32] suggest that dissipation due to fluid expulsion by the growing patch is dominated by fluid velocity gradients in a membrane wedge just outside the patch, and that increasing γ increases resistance by decreasing the wedge angle. This assumption may not be generally applicable but it is conceivable that the conditions required for this result's validity may be realized. We show below that using the alternative, non-linear local velocity-density relation of eq. S14 modifies the exponents in our predicted power law relations but otherwise leaves all conclusions qualitatively unchanged.

Adhesion Kinetics

The general forms of the key time and length scales are unchanged, and the same general expressions for the interfacial complex density Γ_c^* remain valid: $\Gamma_c^* = \Gamma_{\rm snare}$ in the slow regime and the expressions of eqs. S9 and S10 in the intermediate and fast regimes, respectively. Substituting into these the new non-linear local velocity-density relation eq. S14 leads to new growth laws:

$$\frac{v_{\rm patch}}{\widetilde{v}_0} = \begin{cases} \left(\Gamma_{\rm snare}/\widetilde{\Gamma}_0\right)^{3/2}\ , & \Gamma_{\rm snare} < \Gamma_{\rm crit}^1,\ \text{slow} \\ \epsilon^{1/2}\,\lambda^{1/4}\left(\Gamma_{\rm snare}/\widetilde{\Gamma}_0\right)\ , & \Gamma_{\rm crit}^1 < \Gamma_{\rm snare} < \Gamma_{\rm crit}^2,\ \text{intermediate} \\ \lambda^{3/8}\left(\Gamma_{\rm snare}/\widetilde{\Gamma}_0\right)^{3/4}\ , & \Gamma_{\rm snare} > \Gamma_{\rm crit}^2,\ \text{fast}\quad . \end{cases} \tag{S15}$$

Here $\widetilde{\Gamma}_0$ and $\widetilde{v}_0$ are characteristic density and velocity scales and the parameter λ is a dimensionless measure of SNARE binding reactivity,

$$\widetilde{\Gamma}_0 = \frac{\gamma}{kT}\ , \qquad \widetilde{v}_0 = \frac{\gamma}{\eta}\ , \qquad \lambda = k_{\rm snare}\,D_c\left(\frac{\widetilde{\Gamma}_0}{\widetilde{v}_0^2}\right)\quad . \tag{S16}$$

The three growth regimes are separated by critical SNARE density values

$$\Gamma_{\rm crit}^1 = \epsilon\,\lambda^{1/2}\,\widetilde{\Gamma}_0\ , \qquad \Gamma_{\rm crit}^2 = \epsilon^{-2}\,\lambda^{1/2}\,\widetilde{\Gamma}_0\quad . \tag{S17}$$

The result for the slow regime was previously calculated in ref [32].

Figures S1 *a* and *b* show exact numerical solutions for patch growth velocity as a function of SNARE density in the slow/intermediate and fast regimes, respectively. Similarly to Fig. 5 for the linear relation, when velocity and density are appropriately scaled with critical values data for a range of ϵ and μ values collapses onto universal curves. The new power laws are confirmed by the numerical results.

Fusion Kinetics

The form of the fusion probability distribution is unchanged from the form of eq. 15 (main text) plotted in Fig. 6, and the expression of eq. 16 (main text) for the mean fusion time T_{fusion} in terms of v_{patch} and Γ_{snare} remains valid. Using in this expression the new patch velocity-SNARE density results of eq. S15 gives

$$\frac{T_{\text{fusion}}}{\widetilde{\tau}_0} = \begin{cases} \left(\widetilde{\Gamma}_0/\Gamma_{\text{snare}}\right)^{4/3} , & \Gamma_{\text{snare}} < \Gamma^1_{\text{crit}},\ \text{slow} \\ \epsilon^{-1/3}\,\lambda^{-1/6}\left(\widetilde{\Gamma}_0/\Gamma_{\text{snare}}\right) , & \Gamma^1_{\text{crit}} < \Gamma_{\text{snare}} < \Gamma^2_{\text{crit}},\ \text{intermediate} \\ \lambda^{-1/4}\left(\widetilde{\Gamma}_0/\Gamma_{\text{snare}}\right)^{5/6} , & \Gamma_{\text{snare}} > \Gamma^2_{\text{crit}},\ \text{fast} \end{cases} \tag{S18}$$

where $\widetilde{\tau}_0 = 0.88\,(\tau_{\text{fus}}/\widetilde{v}_0^2\,\widetilde{\Gamma}_0)^{1/3}$.

These results apply provided steady state patch growth is reached and many SNAREs accumulate in the patch before fusion is triggered. These requirements are met if eq. 18 of the main text is satisfied. For low enough SNARE density eq. 20 of the main text is satisfied and fusion kinetics are described by the exponential distribution for single-SNARE fusion.
