# Supplementary figures and images for: Model of SNARE-Mediated Membrane Adhesion Kinetics
Source: PLoS One. 2009 Aug 3;4(8):e6375. doi: 10.1371/journal.pone.0006375 (PMC2715897; doi:10.1371/journal.pone.0006375)

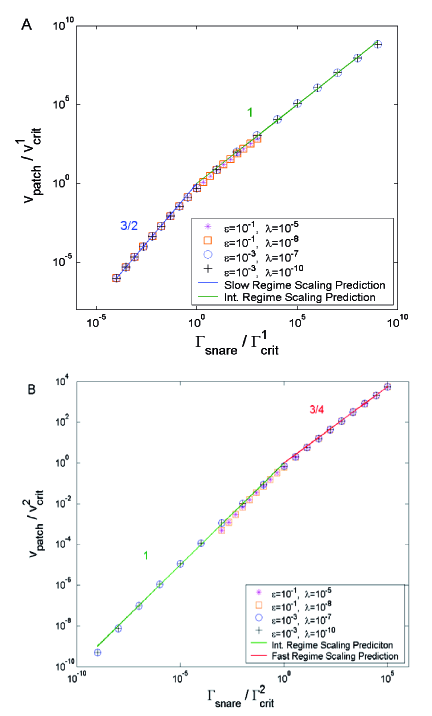

Supplement: Figure S1 — Collapse of scaled SNARE-mediated adhesion data onto a single universal patch growth law. Same as Fig. 5 of the main text, but using non-linear relation between velocity and patch boundary complex density, eq. S19. Symbols indicate exact numerical solutions of patch growth model for a range of parameter values as shown. Solid lines denote scaling predictions. (A) Patch velocity versus SNARE density in slow and intermediate regimes. Density scaled by Γ1 crit and velocity scaled by v1 crit = ε3/2λ3/4v0. (B) As for (A), but for intermediate and fast regimes. Densities and velocities scaled, respectively, by Γ2 crit and v2 crit = ε-3/2λ3/4v0. Numerical results confirm the asymptotic solutions with relative errors in the velocity peaking at the critical densities (106% in (A) and 54% in (B)) and approaching zero far from the critical densities in each regime. (0.64 MB TIF) [file pone.0006375.s002.tif]
